# Tribological Performance of Glass/Kevlar Hybrid Epoxy Composites: Effects of Pressurized Water-Immersion Aging Under Reciprocating Sliding Wear

**DOI:** 10.3390/polym17212944

**Published:** 2025-11-04

**Authors:** Mehmet İskender Özsoy, Mustafa Özgür Bora, Satılmış Ürgün, Sinan Fidan, Erman Güleç

**Affiliations:** 1 Department of Mechanical Engineering, Faculty of Engineering, Sakarya University, Sakarya 54050, Türkiye; 2 Department of Airframe and Powerplant Maintenance, Faculty of Aeronautics and Astronautics, Kocaeli University, Kocaeli 41001, Türkiye; ozgur.bora@kocaeli.edu.tr (M.Ö.B.); sfidan@kocaeli.edu.tr (S.F.); 3 Department of Aviation Electrics and Electronics, Faculty of Aeronautics and Astronautics, Kocaeli University, Kocaeli 41001, Türkiye; urgun@kocaeli.edu.tr; 4 Otokar Automotive Defense Industry Corp., Sakarya 54580, Türkiye; egulec@otokar.com.tr

**Keywords:** reciprocating wear, pressurized water immersion, Kevlar/glass fiber laminates, coefficient of friction, 3D wear profilometry

## Abstract

This study quantifies how pressurized water immersion alters the reciprocating sliding behavior of glass and Kevlar woven fabric-reinforced polymer hybrid composite laminates. Specimens were immersed in deionized water at 10 bar and 25 °C for 0, 7, 14, and 21 days, then tested against a 6 mm 100Cr6 steel ball at 20 N under four regimes that combine 1 or 2 Hz with 10 m or 20 m total sliding. Water uptake rose from 0 to 8.54% by day 21 and followed a short-time Fickian square root of time trend, indicating diffusion-controlled sorption. The coefficient of friction exhibited a robust nonmonotonic response with a pronounced minimum at 14 days that was typically 20 to 40% lower than the unaged reference across frequencies and distances, while 7 days produced a partial decrease and 21 days trended upward. Three-dimensional profilometry showed progressive widening and deepening of wear tracks with immersion, for example, at 1 Hz and 10 m width increased from about 1596 to about 2050 to 2101 μm and depth from about 128 to about 184 to 185 μm, with a transient narrowing at 2 Hz after 7 days. Scanning electron microscopy corroborated a transition from mild plowing to matrix plasticization with fiber–matrix debonding and debris compaction. Beyond geometric wear metrics, this study re-processed the existing profilometry and COF records to derive a moisture-dependent mechanistic approach. Moisture uptake up to 8.54% reorganizes the third body at the interface so that friction drops markedly at 14 days (typically 20–40% below the unaged state), while concurrent matrix plasticization and interface weakening enlarge the wear cross-section extracted from the same 3D maps, decoupling friction from damage width/depth under wet conditioning. Factorial analysis ranked immersion time as the dominant driver of damage for width and depth with frequency as a secondary factor and sliding distance as a minor factor, highlighting immersion-controlled tribological design windows for marine and humid service.

## 1. Introduction

Today, fiber-reinforced composites have found applications ranging from everyday use to aircraft due to their specific strength, fatigue life, and corrosion resistance [1,2,3,4,5,6]. However, despite their widespread application, the combined influence of environmental exposure and tribological loading remains insufficiently explained, particularly when water immersion alters both the chemical structure of the polymer matrix and the interfacial load distribution between fibers. To overcome specific deficiencies, hybrid composites, which integrate two or multiple different fiber types within a single polymer matrix, have attracted interest in research studies. This design produces a synergistic “hybrid effect” that allows tailored properties and, in principle, cost reduction by hybridizing valuable fibers with less expensive ones [7,8,9,10,11]. Polymer composites can be reinforced with different types of fibers such as carbon, glass, and Kevlar. To obtain new properties, hybridization merges different fibers within a single fabric material. Woven fabrics increase resistance against the propagation of cracks, as the weft and warp threads resist opening of the matrix through brake opening restriction. Their interlocking design also minimizes material anisotropy. Hybrid woven fabrics, which integrate through methods such as the univalent use of glass and carbon-based hybrid materials, therefore synergistically integrate their superior traits within a distinct material [12]. The expensive nature of the carbon- and Kevlar-based fibers demands hybridization with the less expensive materials such as glass fibers. This results in a composite possessing superior strength, stiffness, fracture toughness, and shock resistance with overall weight and cost reduction. The optimization of the mechanical properties and reduced cost of the resultant material leads to increasing demand for hybrid materials that is sternly pushing their use rapidly across advanced applications such as aerospace, automobile, defense, naval ship, and medical equipment fields [13].

Literature review finds that no studies have addressed hybridization of identical E-glass and aramid fiber fabrics. These composite elements have the potential as substitute materials for special aircraft elements, such as in aircraft and gliders. In addition, such advanced hybrid composites can ideally replace conventional metallic elements in critical parts of helicopters, for example, the main shaft and tail rotor blades. Scientific research on such composite elements for such applications is thus crucial [14]. The hybrid combinations that are popular are as follows: glass–aramid, glass–carbon, and carbon–aramid hybrid combinations. Though there is an ample amount of literature on the mechanical properties of such hybrids, tribological performance is rarely considered. The aerospace industry develops carbon–aramid hybrids as alternatives for conventional carbon-based composites. Given that the components usually function under wear-loading conditions, resistance to wear is paramount. Among these, glass/Kevlar hybrid laminates are particularly promising for aerospace and marine components, yet their long-term wear durability under hydrothermal conditions has been studied far less systematically than their static mechanical behavior. The MAF and MCF were obtained by self-assembling the chitosan. The technique greatly enhanced the interfacial adhesion between fiber and epoxy, with MAF/EP and MCF/EP forming with 15.61% and 18.06% improvement in IFSS, correspondingly. The performance of the MCF/EP coating surpassed that of MAF/EP with 11.09 increased tensile strength, but it reduced the wear rate by 66.35%. In addition, it had an elevated load bearing with interfacial strength that reduced erosion volume by 50.38%; this is due to its superior resistance of SiC particles from the particle of the carbon fiber [15]. Nevertheless, most of these studies primarily focused on mechanical retention properties such as tensile or flexural strength and did not extend their observations to surface degradation mechanisms under sliding contact. Although some reports highlighted moisture absorption kinetics and fiber–matrix debonding [12,13,14,15], they did not correlate these effects with wear morphology or frictional behavior. This lack of integrated mechanical–tribological analysis represents a significant gap in the current understanding of hybrid laminate durability.

Hriberšek and Kulovec [16] examine the durability and thermal performance of steel/polymer gearbox pairs. The polymers, which were based on polyacetal (POM) and polyamide 66 (PA66), were filled with aramid or carbon fibers and PTFE-based internal lubrication. Findings show that POM and carbon-reinforced PA66 gears had the greatest durability with the lowest coefficient of wear. High correlation was evident between the meshing temperature and flank wear progression and thus verified thermal response as a significant factor for gearbox performance and selection material choice. Çetin et al. [17] produces carbon–aramid hybrid composites from a halloysite nanotube (HNT)-substituted epoxy matrix material. Ball-on-disk studies showed that adding HNT decreased the friction coefficient by 9–11% and reduced wear rate by 75% versus neat epoxy. Three-dimensional topography and thermal imaging indicated improved surface form and heat dissipation; SEM/EDX pointed to an HNT-derived tribofilm as the primary mechanism.

Composite materials have received considerable interest over the past few decades, both from academic and other fields, due to their distinct mechanical properties and advantages. Although composite materials have corrosion resistance, physical and chemical structures of composite materials for different environments alter with environmental conditions. Therefore, the contribution of environmental conditioning such as hygrothermal and hydrothermal and thermal and ultraviolet aging on long-term performance of fiber-reinforced composite materials remains an external concern [18]. When polymer matrix composite materials come in contact with the marine environment and climatic conditions such as high temperature, humidity, and ultraviolet radiation, deterioration of material properties is noticed that badly impacts their performance [19]. Erdoğan and Demirbaş [20] study the thermomechanical performance of Carbon–Basalt–Aramid (C-B-A) and Glass–Basalt–Aramid (G-B-A) hybrid composites without and with aging (12 h and 48 h). From the results of the thermomechanical analysis (TMA), it was noticed that their properties were completely changed by the presence of aging significantly. For C-B-A aged composite materials, dimensional change and coefficient of the thermal expansion (α) were enhanced by 2.3 and 1.85 times, and the glass transition temperature (Tg) reduced by 0.855 times from the unaged material’s value. The changes for the G-B-A composite materials were deeper, and the dimensional change and α enhanced by 2.44 and 2.24 times, and the value of Tg reduced 0.93 times from the unaged material’s value. The obtained results indicate the great contribution of the effect of aging and selection of reinforcement on hybrid composite performance. Çiçek et al. [21] studies the so-far unreported internal failure pressure of four-layer composite pipes having hybrid glass/aramid fiber reinforcement. Pipes have been fabricated with four different layer arrangements such as all glass, and hybrid with single and double layers of aramid located at different intermediate locations. From the test results, it was noticed that failure pressure highly depended on the type of reinforcing architecture. The lowest damage pressure value was noticed in all-glass fiber samples. On the other hand, the hybrid configuration having two layers of glass and two layers of aramid fiber configuration provided maximum failure pressure value and noticed the improvement due to this specific hybridization. Liao et al. [22] studies a broken-down GFRP pipe from a 15-year-old sour oilfield. Comparative study between degraded and healthy zones disclosed serious defects, such as voids, reduced resin content, and extreme under-curing in the outer layer. The chemical deterioration of the amine curing agent and oxidation of resin at the fiber–matrix interface were detected by spectroscopic analysis (FTIR, XPS). The results were corroborated by nanoindentation that presented extreme decrease in nanohardness and elastic modulus of degraded outer layer. While these findings clarify frictional responses for single-fiber composites, their results cannot be directly generalized to hybrid systems, where differences in fiber stiffness, interfacial chemistry, and water absorption lead to more complex wear mechanisms.

Tian et al. [23] designed a multi-filler reinforced epoxy composite (MFREC) for steel protection coatings. When subjected to hygrothermal aging, water absorption proceeded with a two-stage diffusion mechanism. The degradation of properties was due to resin hydrolysis and de-bonding at the filler/matrix interface. Despite the reduction in interfacial shear strength between the steel and fillers, the fillers dispersed managed to prolong the trajectory of the crack and resisted complete failure in the process of bonding. For one, MFREC showed outstanding wear resistance, especially with lubricants such as water. However, hygrothermal aging highly decreased its anti-wear ability such that the specific wear rate rose by over 255% following 60 °C immersion. Bandaru et al. [24] observed the impact of hygrothermal aging (35 °C distilled water, 60 days) on the abrasive wear of glass/PTFE composites with and without non-hybrid and steel mesh (MM) hybrid variants. The specimens were tested against SiC papers with loads of 10–50 N. The aging proceeded with Fickian diffusion, and DSC study identified reduced crystallinity with enhanced water absorption. Hygrothermal aging lowered the abrasive wear resistance of all the composites with reference to non-aged samples. Nevertheless, under equivalent aged conditions, the use of a layer of a steel mesh (MM) enhanced the wear resisting ability of non-hybrid composites tested. Sayalık and Temiz [25] considered the dry sliding wear for the aging period of 30–90 days of the carbon/epoxy (CFRP) and the glass/epoxy (GFRP) polymer matrix composites subjected to artificial seawater, engine oil, and diesel immersion mediums. Test specimens prepared with the VARTM process were examined with the pin-on-disk setup according to ASTM G99-17 with a hardened steel disk against 10–30 N loads. The results stipulated that the environment of aging critically contributed towards influencing the wear. Low weight removal was observed following diesel exposure, and engine oil provided maximum material removal. On the contrary, the seawater-aged samples provided the least contribution towards the specific wear rate such that there was a different degradation mechanism with reference to the hydrocarbon-based constraint fluids. Pradhan et al. [26] examines the four-week natural seawater durability of GO-dispersed GFRP composites. Both conventional and 0.5 wt% GO-GFRP samples were prepared and characterized. The interlaminar shear strength (ILSS) reduced by 35% for conventional GFRP but just 24.8% for the GO-reinforced composite after seawater aging. In the same way, the glass transition temperature (Tg) reduced by 16% and 15.2%, correspondingly. Fractographic examination performed using a scanning electron microscope (SEM) presented that the hybrid composite’s superior performance is due to superior fiber–matrix interfacial bonding enhanced by the GO filler, which suppresses hygrothermal degradation. Qin et al. [27] surveys the durability of fiber-reinforced composites that degrade due to environmental aging parameters such as temperature and humidity. It condenses microscopic aging mechanisms and examines property variations in tensile, bending, and shear strength in seven types of climate ranging from tropical desert climate to seawater immersion. Differing models such as the Arrhenius model and residual property-based models have been discussed. To optimize aging life and service life prediction accuracies, future models need to incorporate service-realistic mechanical loads, integrate information from different climates, and apply comprehensive property indices and reliable natural vs. accelerated aging equivalencies achieved from standardized databases on accelerated testing rather than from hypothetical models assumed on short-term test results at specific temperatures. Ahmad et al. [28] examines the hygrothermal degradation of glass/bamboo fiber-reinforced epoxy composites filled with nanoclay. Water immersion lowered the tensile and flexural strengths for all the composites by 7.4–14.8% and 7–13.6%, correspondingly. Nevertheless, the addition of nanoclay suppressed this degradation, raising the dry-state tensile and flexural strengths by 6–11% and downward the strength reduction on soaking by 7.4–12% and 7–11%, respectively. Scanning electron microscope (SEM) fractography validated the role of nanoclay for superior interfacial bonding and moisture uptakes reduction and thus enhanced the durability of the composite material. Velmurugan and Natrayan [29] examine the cryogenic strength at 77 K of the hybrid glass/Kevlar fiber composites for naval applications, where Kevlar compensates for the tendency of glass fiber-reinforced composites for degradation due to saltwater aging. The cryogenic mechanical properties for five stacking sequences were put under test and examined. Hybrid composites presented superior performance without failure at a catastrophic scale. Type II (G-G-K-K-G-G) possessed maximum tensile strength (333.25 MPa), and Type IV (K-G-G-G-G-K) possessed maximum flexural strength (460.58 MPa) and impact strength (152.11 kJ/m^2^). 30 min cryogenic treatment possessed optimum mechanical properties as compared to 15 and 45 min exposure time. The results verify that hybridization works optimally for durability improvement for severe environments. Muralidharan et al. [30] examine carbon/glass (CG) and kevlar/glass (KG) hybrid matrix systems for naval applications, with special reference to seawater diffusivity and retention of tensile strength on hydrothermal aging (20–60 °C for 300 days). Maximum moisture absorption behaved at 60 °C over 300 days, and lowest tensile strengths were obtained after a 150-day aging period. Significantly, both hybrid systems still managed and held a considerable percentage of strength, with CG and KG composites pegging that they will have 75 and 70 percent maximum tensile strength, respectively, at 40 °C for 100 years. These results confirm the promising long-term durability for long-term applications in marines and in the outdoors for such hybrid matrix systems.

Hassan et al. [31] compares the abrasion and mechanical performance of Kevlar, carbon fabrics, and their epoxy composites under dry, wet, and simulated sweat conditions. The tensile strength of Kevlar fabric under dry conditions was the highest, but overall mechanical performance was better for carbon/epoxy composites due to better adhesion between the matrix and fiber. Hybrid composites provided a balanced performance. Abrasion resistance was maximum for Kevlar but increased in wet conditions for all materials, with the maximum value in the corrosive salt solution for each material. These findings are relevant to body protection and aerospace applications and demonstrate the necessity for protection from hygrothermal degradation and for developing long-lasting and sustainable composites for stringent service applications. Intra-ply hybrid composites of kevlar and hemp fibers with plain, basket, and twill weaves were prepared through compression molding. Hygrothermal test results indicated that the hemp composites absorbed maximum water with 8.45% and swelled to maximum thickness with 4.34 value, but Kevlar and pure epoxy foils presented maximum resistance. The hybrid performance was all intermediate and confirmed effectiveness for hybridizing. Solid particle erosion test results, following Taguchi design guidance, stated the best performance for the basket weave hybrids showing minimal erosion at 90° impact angle for 2 min elapsed time as the optimum configuration. Micro-cavities, fiber failure, and microcracks due to morphological analysis of the erosion surfaces led to identifying the major mechanisms of wear [32]. Based on the literature review conducted, it can be observed that hybrid fiber-reinforced composites that use glass, carbon, and aramid fibers have been studiously investigated for their mechanical performance, environmental durability, and partly for their tribological performance under dry or lubricated conditions [19,20,21,22,23,24,25,26,27,28,29,30,31,32]. Despite extensive prior work, the combined influence of hygrothermal aging and reciprocating wear in glass/Kevlar hybrid laminates has been less systematically examined. Prior studies primarily addressed tensile/flexural degradation, moisture diffusivity, and long-term performance; only a few investigated sliding wear under environmental conditioning. Therefore, a systematic investigation combining water immersion with reciprocating sliding is necessary to clarify how hydrothermal conditioning modifies both frictional and morphological responses. Such analysis would bridge the gap between previous mechanical aging studies and tribological performance evaluations. This gap is relevant to marine and aerospace structures where long-term water exposure and cyclic friction coincide. This study investigates glass/Kevlar–epoxy laminates conditioned in 10 bar waters for 0–21 days, quantifies water uptake, and evaluates reciprocating wear (1–2 Hz; 10–20 m) using 3D profilometry, ANOVA, and SEM. The water uptake was directly measured, and reciprocating sliding wear tests were conducted at two regimes (1 Hz and 2 Hz) and sliding distances (10 m and 20 m). The results were extensively examined through evolution of coefficient of friction (COF) and wear track width and depth, supported with three-dimensional profilometry. In addition, statistical analysis based on ANOVA allowed prominent parameters affecting wear response to be ascertained, whereas SEM characterization provided the associated underlying wear mechanisms instigated by both hygrothermal and reciprocal frictional loading instigations. This study clearly integrates moisture uptake, frictional response, and damage evolution by post-processing data already acquired; COF histories, 3D profilometry, and SEM. This integrated analysis reveals why an intermediate immersion (14-day) minimizes µ yet increases the wear cross-section, establishing moisture-controlled design windows for humid and marine service.

## 2. Materials and Methods

### 2.1. Materials

**Figure 1:**
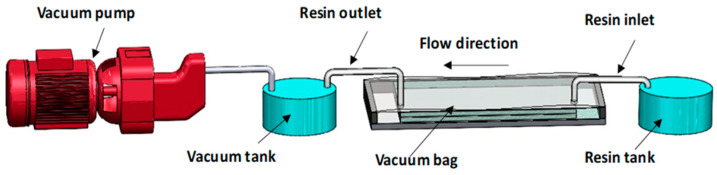
Schematic illustration of the vacuum infusion process.

In this study, polymer matrix was reinforced with aramid and glass fibers. The glass fabric used was of areal density 500 g/m^2^, whereas the woven type of Kevlar fiber has an areal density of 400 g/m^2^. Fiber reinforcements were provided by KP Kompozit Pazarı Inc. Istanbul, Türkiye. The composite laminate was planned with eleven layers in the hybrid stacking sequence of [K/G/K/G/K/G/K/G/K/G/K]. Sika CR80 epoxy resin and CH80-2 hardener set (Sika Österreich GmbH, Bludenz, Austria) was selected as the matrix material. The composite laminates were manufactured using the vacuum infusion method, which ensures uniform resin distribution, reduced void content, and improved fiber wet-out. In this process, the dry fiber fabrics were placed on the mold surface in the predetermined stacking sequence, followed by the placement of peel ply, flow mesh, and vacuum bagging film. A controlled vacuum was applied to facilitate resin infiltration through the fiber network, ensuring complete impregnation of the reinforcement layers. After resin infusion, the laminates were left to cure under ambient conditions, followed by post-curing to achieve optimal mechanical properties. Figure 1 shows the schematic illustration of the vacuum infusion process.

### 2.2. Experimental Setup

**Figure 2:**
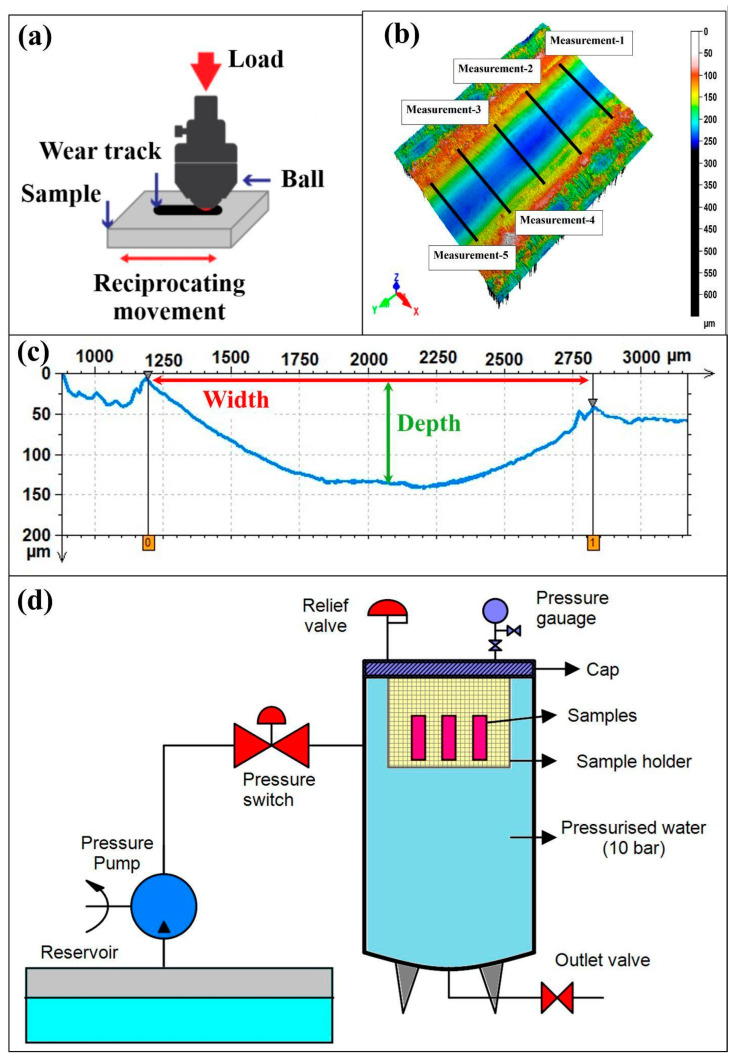
Experimental setup used in reciprocating wear tests and water-immersion aging: (**a**) reciprocating wear test setup, (**b**) wear track 3D measurements taken from reciprocating wear track, (**c**) wear track width and depth measurements taken from reciprocating wear track, (**d**) pressurized water aging test setup.

Reciprocating wear tests were carried out on hybrid epoxy composites after water immersion for 0, 7, 14, and 21 days. Using the UTS Tribolog™ Multi-Function Tribometer (Istanbul, Türkiye) in reciprocating mode (Figure 2a, a stationary 6 mm 100Cr6 steel ball (60–66 HRC; as-supplied Ra < 0.05 µm) was pressed against the flat composite coupon under a constant normal load of 20 N while the specimen oscillated linearly to generate an elongated wear scar. Normal loads in the 10–20 N range for a 6 mm 100Cr6 counter-face yield Hertzian contact pressures characteristic of polymer–steel reciprocating contacts; in this study 20 N was therefore selected as the primary load to ensure a measurable wear response without inducing gross subsurface damage.

Tests were executed at stroke frequencies of 1 Hz and 2 Hz to total sliding distances of 10 m and 20 m, yielding four parameter sets: 1 Hz–10 m, 1 Hz–20 m, 2 Hz–10 m, and 2 Hz–20 m (Table 1). The 1–2 Hz and 10–20 m settings effectively balance frictional self-heating and cycle count, enabling the capture of steady-state behavior over hundreds of strokes while minimizing thermal or oxidative confounders. Composite coupons had an initial surface roughness of Ra = 3 µm; before each run, both counter-face and specimens were ultrasonically cleaned in high-purity isopropanol and dried with oil-free nitrogen, and a brand-new ball was mounted to avoid cross-contamination and pre-existing transfer films. Coefficient-of-friction (COF) time histories were acquired at 1000 Hz and, for visualization only, down-sampled to 10 Hz by non-overlapping 100-point boxcar averaging with additional stroke-wise cycle averaging, with no further filtering or baseline correction. All four reciprocating parameter combinations were performed at each immersion interval.

Figure 2b presents a representative three-dimensional surface map of a reciprocating wear scar acquired with a non-contact laser profilometer; five evenly spaced transverse analysis windows (measurement 1–5) were placed across the track to compute local scar width and depth, and the mean of these values with their scatter provided the wear metrics and percentage error bars. Figure 2c shows the corresponding transverse line profile extracted from the profilometric dataset, from which wear track width (shoulder-to-shoulder span) and maximum depth (valley relative to the unworn baseline) were obtained; replicate profiles taken at equidistant positions along the stroke were averaged and their dispersion was reported as uncertainty to capture any longitudinal heterogeneity.

**Figure 3:**
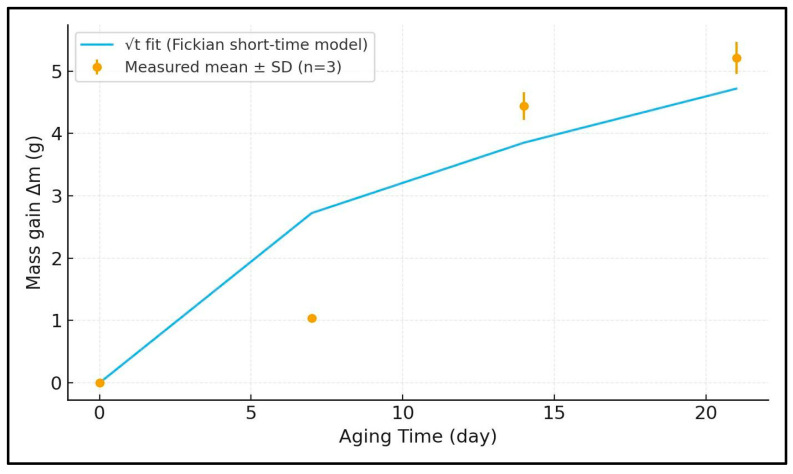
Mass gain (Δm) versus water-immersion time for the glass/Kevlar epoxy hybrid composite reinforced (*n* = 3, mean ± SD), with a √t Fickian short-time fit superimposed, evidencing rapid early uptake and a gradual approach toward quasi-equilibrium by day 21.

Pressurized water-immersion aging was performed in a closed vessel filled with deionized water at 25 ± 1 °C and regulated to 10.0 ± 0.1 bar by a pump/pressure-switch loop with an inline relief valve and gauge; composite coupons mounted on a PTFE holder were immersed for 0, 7, 14, or 21 days, then, after depressurization, gently surface dried and conditioned for 24 h at 23 ± 2 °C and 50 ± 5% RH prior to tribological testing (Figure 2d). An immersion pressure of 10 bar was adopted to emulate submerged/pressurized service and to accelerate uptake at 25 ± 1 °C without cavitation or thermal artifacts, within the diffusion-controlled regime. The conditioning intervals of 7 days, 14 days, and 21 days were chosen to sample the transient, intermediate, and near-equilibrium segments of the uptake curve; in our laminates the measured mass gains were 1.70%, 7.28%, and 8.54%, respectively, with 14 days–21 days approaching quasi-saturation (Figure 3).

### 2.3. Data Post-Processing and Derived Tribological Metrics

Wear cross-sectional area *Acs* was computed from the existing transverse line profiles (Figure 2c), and wear volume per unit sliding distance *V/L* was estimated as *Acs* times the stroke coverage factor applicable to reciprocating motion.

A specific wear rate in the Archard sense was then expressed as

(1) $$k=\frac{V}{N\;L}$$

using the already reported normal load *N* and track length *L*.

**Figure 4:**
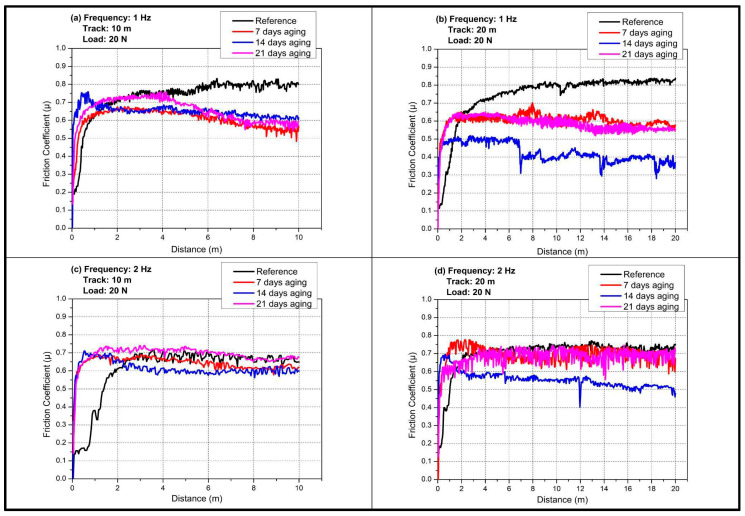
Effect of aging time on COF values at 20 N load in reciprocating wear tests: (**a**) 1 Hz; 10 m; (**b**) 1 Hz; 20 m; (**c**) 2 Hz; 10 m; (**d**) 2 Hz; 20 m.

In parallel, a frictional energy per unit sliding distance was estimated as *E = μN* using the measured steady-state COF plateaus for each condition (Figure 4), allowing us to compare energy input with damage growth without additional experiments. All calculations use the profilometry/COF datasets already collected.

## 3. Results

### 3.1. COF Analysis of Reciprocating Wear Tests

Water uptake of the glass/Kevlar hybrid epoxy laminates were measured at 0, 7, 14, and 21 days and are reported as mean ± SD over three replicates (error bars in the figures; SD ≤ 5% of the mean at all times). The average mass gains were Δm(0) = 0.000 g, Δm(7) = 1.038 ± 0.052 g, Δm(14) = 4.440 ± 0.222 g, and Δm(21) = 5.210 ± 0.261 g, corresponding to normalized uptakes M(t) = Δm/m_0_ of 0, 1.70%, 7.28%, and 8.54% for m_0_ = 61.003 g. The small dispersions (CV ≤ 5%) indicate good reproducibility and a uniform conditioning state across coupons. A least-squares fit to a short-time Fickian model (Δm ∝ √t) captures the rise and the gradual approach toward a plateau, supporting a diffusion-controlled sorption mechanism. The Kevlar-containing interphases, known to be more hydrophilic than glass/epoxy, plausibly provide additional ingress pathways at intermediate times, whereas the glass fraction and the woven architecture limit swelling and slow the approach to saturation. The decelerating increment from 14 to 21 days suggests the system is nearing, but has not yet reached, equilibrium under the present exposure. Accordingly, tribological performance is strongly governed by swelling duration up to the quasi-equilibrium condition (14 days), at which the COF reaches a minimum; further immersion produces only marginal changes as water uptake and matrix plasticization approach saturation (Figure 3 and Figure 4). Quantitative determination of the diffusion coefficient D via Fick’s second law would require the specimen thickness/geometry; nevertheless, the √t scaling together with the low scatter and sub-10% total uptake indicates predominantly Fickian behavior and satisfactory hydrothermal resistance of this hybrid laminate over the tested immersion period.

As shown in Figure 3, the mass gain exhibits a rapid early uptake that follows a √t (Fickian) trend and progressively approaches quasi-equilibrium by day 21. Statistical reviews and meta-analyses of hygrothermal aging data confirm that the early-time sorption of water in epoxy systems is commonly Fickian (Mt ∝ √t) and that reported diffusion coefficients and initial slopes are highly sensitive to resin chemistry, specimen thickness, and test temperature, which explains why a clean √t fit captures the short-time region so well [33]. Thickness-dependent modeling work shows that specimens with the thicknesses typical for laminated composite coupons often exhibit an initial Fickian regime followed by slower approach-to-equilibrium or apparent non-Fickian effects as edge saturation, capillary filling of interfacial voids, and fiber–matrix interfacial storage begin to contribute at longer times, behavior that explains an initially precise √t fit that gradually departs as the sample approaches the 21-day quasi-equilibrium [34]. Finally, topical reviews of moisture uptake in fiber-reinforced composites highlight the role of fiber type (glass vs. aramid/Kevlar), fiber volume fraction, and the integrity of the fiber–matrix interface in setting both the equilibrium uptake and the kinetics: glass/Kevlar hybrid laminates commonly show an early, matrix-dominated Fickian uptake followed by slower, interface-controlled processes (interfacial sorption, microcracking, void filling) that control the long-term plateau observed near three weeks immersion. Together, these studies provide a mechanistic basis for the curve shape observed in Figure 3 (accurate √t short-time fit plus gradual approach to equilibrium by day 21) [35].

Figure 4a shows that at 1 Hz and 10 m the reference rapidly runs in and stabilizes near μ about 0.7–0.75, while immersion depresses and smooths the response, with 14 days giving the lowest plateau near 0.50–0.55 and 7 and 21 days leveling around 0.58–0.65.

Figure 4b indicates that extending the track to 20 m at 1 Hz magnifies these differences because cumulative sliding consolidates a hydrated third body on the intermediate-aged surface, so the 14-day curve remains near 0.40–0.45 across the entire distance, whereas 7 and 21 days drift toward 0.60 and the reference stays highest.

Figure 4c demonstrates that raising frequency to 2 Hz at 10 m shortens the running-in regime and increases frictional energy input, which slightly elevates the plateaus and narrows the gap between the reference and 21 days, yet the 14-day curve is still the lowest and exhibits reduced noise that is consistent with a coherent water-assisted tribofilm.

Figure 4d confirms that the most demanding combination of 2 Hz and 20 m preserves the advantage of the 14-day condition despite occasional transient drops linked to film renewal, while the 7- and 21-day traces approach the reference and show intermittent spikes that suggest partial tribofilm rupture and debris-driven stick–slip, indicating that intermediate immersion provides an optimal balance of epoxy plasticization, interfacial compliance, and moisture-mediated boundary lubrication.

Water immersion time exerts a nonmonotonic influence on COF, with an intermediate conditioning at 14 days producing the most pronounced and persistent reduction in friction, typically 20–40% lower than the dry reference across both frequencies and track lengths, whereas 7 days yields a partial decrease and 21 days trends back upward, especially at 2 Hz and 20 m. This behavior is consistent with a balance between beneficial water-induced plasticization and interfacial hydration that stabilizes a low-shear tribofilm at intermediate uptake, versus over-plasticization, interfacial swelling, and debris-assisted instabilities that emerge at longer immersion and re-elevate COF, with longer sliding distances amplifying these differences. The non-monotonic COF behavior observed in Figure 4, where an intermediate immersion (14-day) produces a persistent, 20–40% lower friction plateau, while shorter (7-day) and longer (21-day) exposures show higher and more unstable COF, is consistent with mechanisms reported in the hygrothermal tribology literature. Hygrothermal conditioning can promote the formation of a coherent, moisture-mediated boundary/third-body film that lowers shear strength at the sliding interface and stabilizes friction during moderate exposure, producing a pronounced reduction in steady-state COF (as reported for multi-filler epoxy systems showing transient tribofilm formation and reduced friction after intermediate aging) [23]. At longer immersion times, however, progressive resin hydrolysis, interfacial debonding and matrix plasticization increase debris production and weaken load-bearing interfaces; these processes both raise effective contact compliance and promote intermittent film rupture and debris-driven stick–slip, which elevates the COF toward or above the dry reference [24]. Moreover, controlled studies on the role of moisture and oxygen show that small changes in environmental water content can switch tribo-chemical pathways and transfer-film character; so, a system can move from moisture-assisted low-shear sliding (low COF) to moisture-aggravated wear (high COF) as hygrothermal damage accumulates, exactly matching the intermediate-optimal/long-term-deterioration pattern recorded in Figure 4 [36].

### 3.2. Moisture–Friction–Damage Integrated Analysis

Placing moisture uptake (Figure 3), and COF plateaus (Figure 4), side by side shows that moisture does not act solely through geometric widening/deepening.

At 1 Hz–10 m, for example, width grows from 1596 μm (unaged) to 2050–2101 μm while depth increases from 128 to 184–185 μm with immersion, whereas the COF simultaneously exhibits a pronounced minimum at 14 days (typically 20–40% below the reference). This decoupling indicates that water-assisted third-body stabilization lowers interfacial shear (μ), yet hydrolytic plasticization and interfacial debonding increase compliance and lateral plowing, expanding the track.

Consistently, the derived k trends increase with immersion and peak beyond 7-day, while the estimated frictional energy per distance E dips at 14 days due to reduced μ. Together with SEM images which transitions from mild plowing to matrix plasticization, fiber/matrix debonding, and debris compaction, these results mechanistically connect moisture content to both sliding shear and damage morphology; intermediate conditioning promotes a coherent, low-shear tribofilm (low μ), whereas longer conditioning degrades the load-bearing interface, raising wear despite μ recovering toward the dry value.

### 3.3. Wear Damage Evaluation

**Figure 5:**
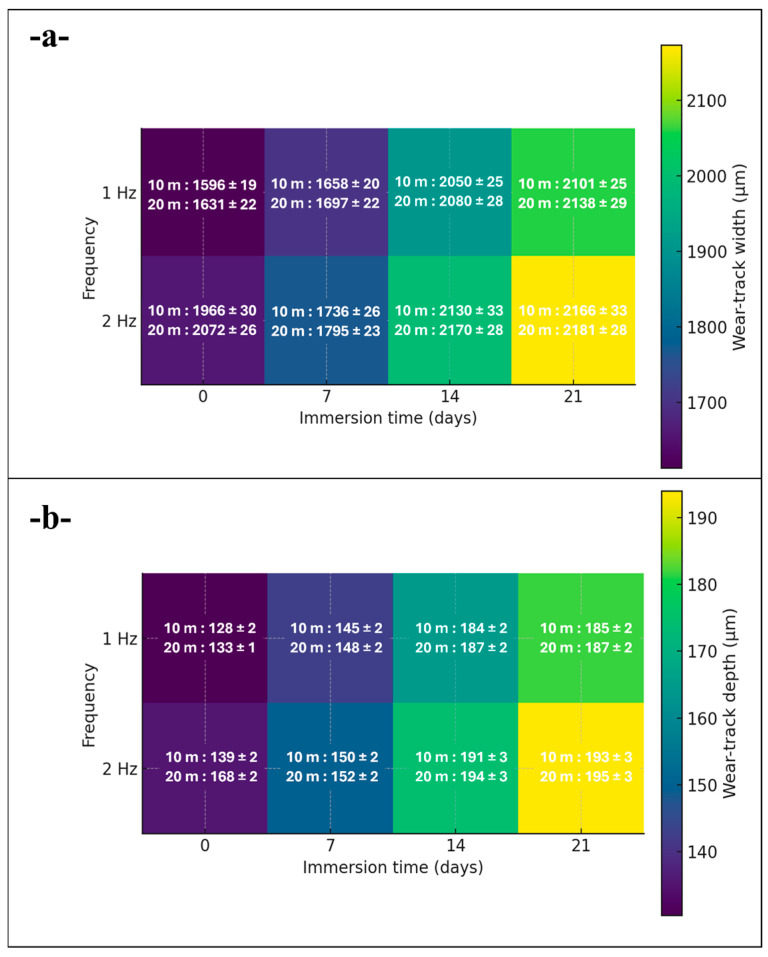
Consolidated heatmaps of wear-track (**a**) width and (**b**) depth versus immersion time (0–21 d) and frequency (1–2 Hz), annotated for 10 m and 20 m sliding distances (mean ± SD, *n* = 3). Trends reveal immersion time as the dominant driver, with frequency secondary and track length minor, aligning with factorial results.

To facilitate an at-a-glance appraisal of global trends, all wear track width (Figure 5a) and depth (Figure 5b) data were consolidated into summary heatmaps across immersion time and frequency (Figure 5). These overviews confirm a monotonic increase with immersion (most pronounced by 14-day) with frequency acting as a secondary amplifier and track length as a minor contributor. 

**Figure 6:**
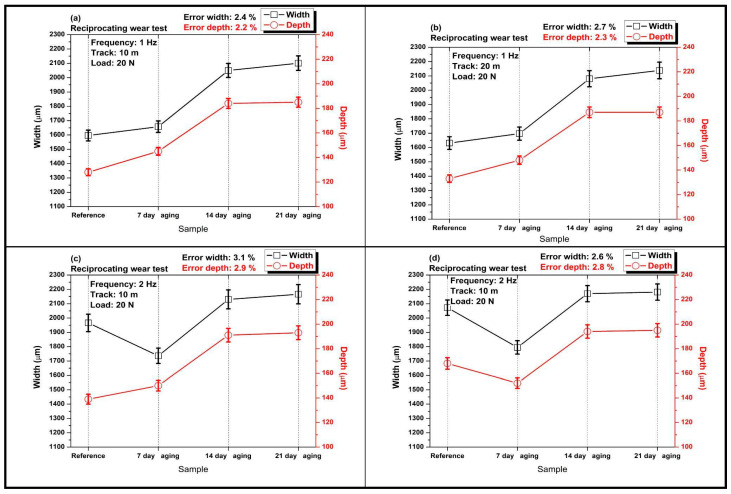
Comparison of width and depth values in reciprocating wear tests at 20 N load with various aging durations: (**a**) 1 Hz; 10 m; (**b**) 1 Hz; 20 m; (**c**) 2 Hz; 10 m; (**d**) 2 Hz; 20 m.

Figure 6 compares the evolution of reciprocating wear track width and depth for the glass/Kevlar epoxy hybrid composites tested at 20 N under four parameter sets: 1 Hz/10 m, 1 Hz/20 m, 2 Hz/10 m, and 2 Hz/20 m, after 0, 7, 14, and 21 days of water immersion.

In Figure 6a at 20 N, 1 Hz, and 10 m total distance, both wear track width and depth increase with immersion time; a slight rise from reference to 7 days is followed by a pronounced jump by 14 days, after which depth plateaus while width continues to climb. This trend indicates that hydrolytic plasticization and interfacial weakening become dominant beyond a short conditioning period, allowing wider lateral plowing while subsurface penetration saturates.

In Figure 6b at the same frequency but 20 m distance, the immersion effect is amplified; width and depth grow markedly up to 14 days and then level off or ease slightly at 21 days. The longer sliding distance magnifies matrix softening and fiber/matrix debonding, producing larger scars, whereas the late-stage moderation of depth suggests partial polishing/compaction of debris once the surface is severely damaged.

In Figure 6c at 2 Hz and 10 m, width exhibits an initial dip at 7 days while depth remains comparatively low, consistent with transient boundary lubrication from absorbed water; by 14 days and 21 days, both metrics recover and exceed the reference, reflecting the transition from lubrication-dominated behavior to moisture-induced degradation (microcracking and interfacial debonding) under higher oscillation rate.

In Figure 6d the combination of 2 Hz and 20 m yields the strongest immersion dependence: both width and depth decrease at 7 days but rise sharply at 14 days and remain high at 21 days, culminating in the largest scars among all conditions. The synergy of higher reciprocation rate and longer sliding distance with hydrothermal damage accelerates mechanical softening and interfacial failure, broadening the track and deepening penetration after prolonged aging. Overall, immersion induces progressive softening and interfacial degradation that widens and deepens the tracks, amplified by higher frequency and longer distance, with only a transient 7-day reduction at 2 Hz (moisture-assisted lubrication/compaction) before damage dominates at 14 days and 21 days.

The surface topography changes documented in Figure 6 are underpinned by measurable chemical and mechanical alterations induced by water uptake rather than being purely morphological phenomena. Water molecules absorbed into the epoxy act as a plasticizer, reducing the glassy modulus and yield strength of the matrix and promoting micro-cracking and reduced cohesive energy in the near-surface region; simultaneously, interfacial water accumulation and hydrolysis processes weaken fiber–matrix adhesion. Mechanically this manifests as reduced matrix resistance to shear and indentation, and increased production of fine debris that can be compacted into a third body. The COF behavior (pronounced minimum at 14 days) therefore reflects a competition: at intermediate uptake the matrix is softened enough to allow a coherent, lubricious tribofilm/compacted debris layer that lowers shear strength and COF, whereas at longer immersion progressive hydrolytic degradation and interfacial debonding increase debris production and three-body abrasion, raising effective contact compliance and wear rates.

The width and depth evolutions reported in Figure 6, namely the progressive widening and deepening with increasing immersion time (pronounced at 14 days and approaching saturation by 21 days) and the stronger effect under higher frequency/longer distance, are consistent with documented hydrothermal aging effects on polymeric composites and polymer–matrix tribological systems. Hygrothermal conditioning can initially promote formation or consolidation of a lubricious transfer/tribofilm (reducing penetration and sometimes narrowing the early wear scar), but with continued uptake the matrix undergoes plasticization and interfacial debonding; these processes increase compliance, facilitate lateral plowing and fiber/matrix pull-out, and produce more and finer debris that promotes three-body abrasive action, all of which enlarge track width and increase maximum depth. Experimental studies across different polymeric systems corroborate this two-stage behavior: hydrothermally aged PTFE-based composites show filler-dependent changes in transfer film and increased wear for fiber-filled systems after aging, consistent with matrix softening and debris-assisted abrasion [37]. Under seawater/saturated lubrication conditions, polyamide (PA12) composites likewise display locally increased track width and depth where matrix softening and particle detachment occur [38]. Hydrothermal fatigue studies on glass–ionomer systems further show that thermally/wet aged samples can develop wider and morphologically different friction tracks (with increased nano/microparticle debris and evidence of matrix cracking), linking aging-induced microstructural changes to larger, more irregular scars [39]. Taken together, these studies support our interpretation of Figure 6: a transition from short-term/partial lubrication effects (transient narrowing/reduced penetration) to dominant long-term hydrolytically driven softening and interfacial failure that widen and deepen reciprocating wear tracks, with the effect amplified by higher oscillation rate and longer sliding distance.

**Figure 7:**
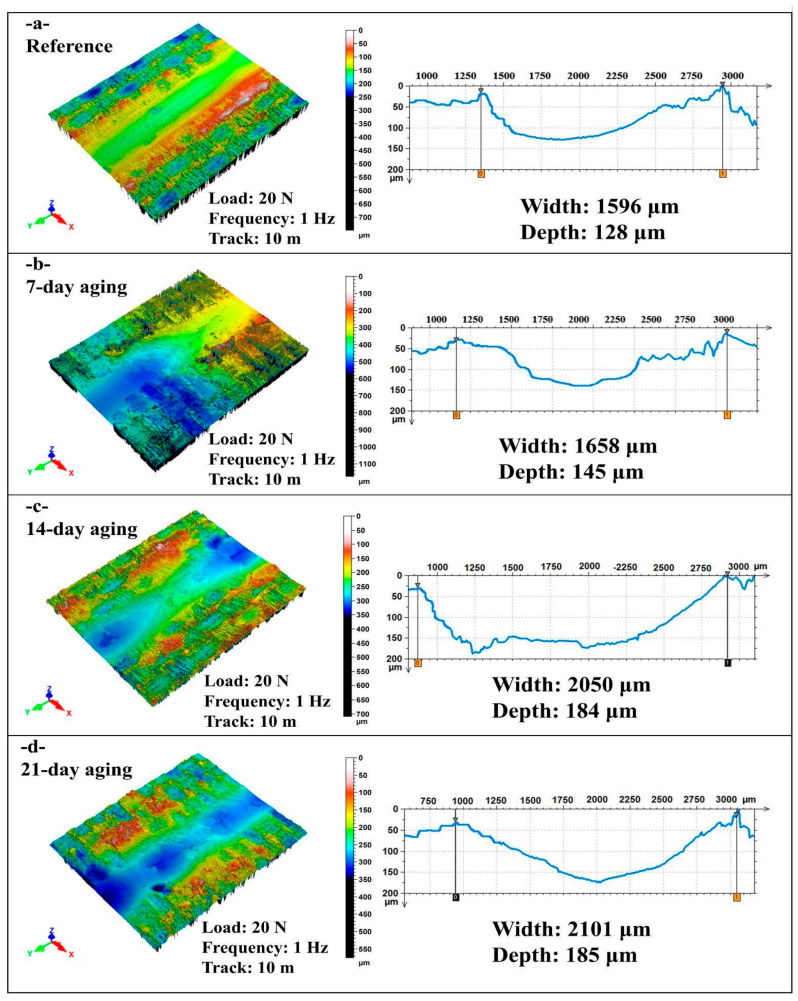
Comparison of width and depth values in reciprocating wear tests at 20 N load, 1 Hz frequency and 10 m sliding distance: (**a**) 0-day aging; (**b**) 7-day aging; (**c**) 14-day aging; (**d**) 21-day aging.

Figure 7 shows non-contact laser profilometry 3D maps and transverse line profiles of reciprocating wear scars for the hybrid epoxy composite tested at 20 N, 1 Hz, and 10 m after 0, 7, 14, and 21 days of water immersion, from which wear track width and maximum depth are quantified.

In Figure 7a under 20 N, 1 Hz, and 10 m, the 0-day (reference) specimen exhibits a relatively shallow reciprocating groove (depth = 128 µm) with a moderate lateral extent (width = 1596 µm); the 3D map shows a continuous valley flanked by modest, symmetric pile-up ridges and a comparatively smooth floor, indicating limited plastic deformation and minimal interfacial failure at the onset of sliding.

In Figure 7b after 7 days of immersion, the track widens slightly (1658 µm, +4% vs. reference) and deepens to 145 µm (+13%), while the topography reveals broadened shoulders and more heterogeneous relief with patches consistent with transfer/debris compaction; these changes point to early-stage moisture-induced plasticization that increases lateral plowing without yet producing catastrophic penetration.

In Figure 7c at 14 days, the wear scar enlarges markedly (width = 2050 µm, +28%; depth = 184 µm, +44%), and the 3D surface shows a deeper, more steep-walled valley with pronounced pile-up and micro-scoring along the reciprocation axis, consistent with matrix softening, fiber/matrix debonding, and easier micro-fracture that together promote both wider material removal and greater subsurface intrusion.

In Figure 7d following 21 days of immersion, the track width and depth remain high (2101 µm and 185 µm, respectively), with the topology displaying a persistently deep groove but a somewhat smoother, compacted floor—evidence of damage saturation where continued moisture-assisted softening maintains large scars while debris polishing moderates further growth in maximum depth.

Water immersion progressively widens and deepens the wear track modestly at 7 days, markedly by 14 days, and then approaching saturation at 21 days, reflecting moisture-induced matrix plasticization and fiber/matrix interfacial weakening that enhance lateral plowing and penetration, with partial debris compaction/polishing limiting further depth growth at the longest exposure.

**Figure 8:**
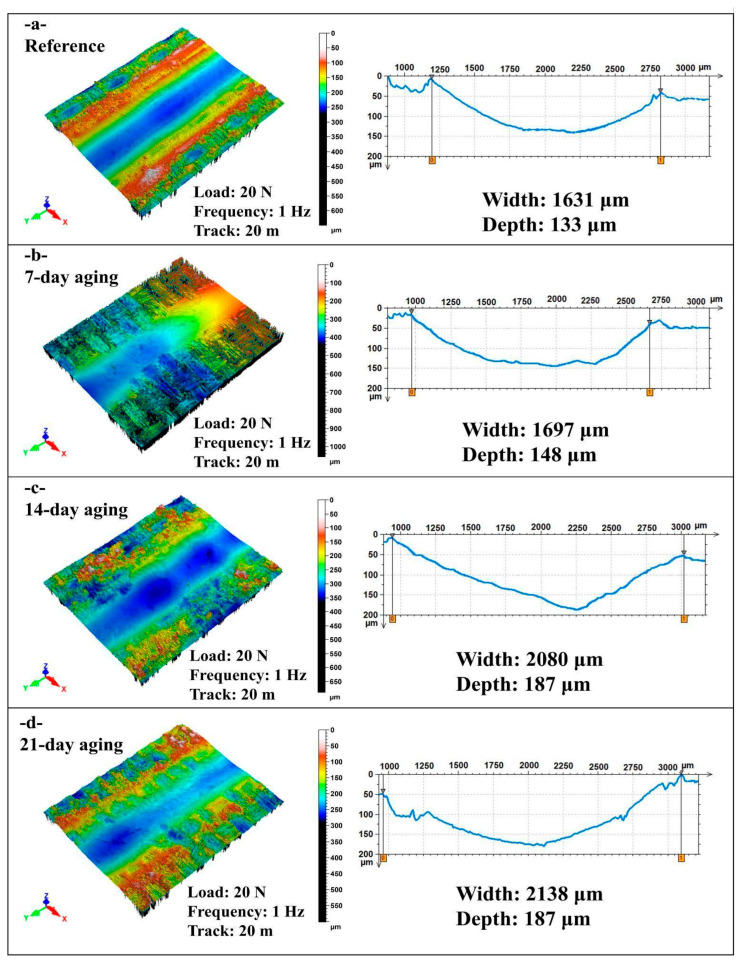
Comparison of width and depth values in reciprocating wear tests at 20 N load, 1 Hz frequency, and 20 m sliding distance: (**a**) 0-day aging; (**b**) 7-day aging; (**c**) 14-day aging; (**d**) 21-day aging.

Figure 8 presents non-contact laser profilometry 3D maps and transverse line profiles of reciprocating wear scars for the glass/Kevlar epoxy hybrid composite tested at 20 N, 1 Hz, and 20 m after 0, 7, 14, and 21 days of water immersion; wear track width and maximum depth are extracted from the profiles.

In Figure 8a at 0-day aging, the track shows a moderately shallow U-shaped valley (width = 1631 µm; depth = 133 µm) with relatively smooth floor and low, symmetric pile-up ridges, indicating limited plastic flow and negligible fiber/matrix disruption at the start of sliding.

In Figure 8b after 7 days, both metrics rise (width = 1697 µm, +4%; depth = 148 µm, +11%), and the 3D topography displays broadened shoulders and more heterogeneous relief with incipient debris compaction—evidence of early hydrolytic plasticization that facilitates lateral plowing and slightly deeper penetration.

In Figure 8c by 14 days, the scar enlarges substantially (width = 2080 µm, +28%; depth = 187 µm, +41% vs. reference); the surface map reveals a deeper, steeper-walled groove with pronounced pile-up and micro-scoring along the stroke, consistent with matrix softening and fiber/matrix debonding that accelerate material removal.

In Figure 8d at 21 days, width increases marginally further (2138 µm), while depth remains at 187 µm, and the groove floor appears somewhat smoother/compacted, suggesting damage saturation where severe moisture-assisted softening maintains a wide track, but continued sliding polishes the valley and limits additional deepening.

Overall, at 20 m sliding distance water immersion progressively widens and deepens the reciprocating wear track, with a pronounced jump by 14 days and a subsequent depth plateau at 21 days; these trends reflect hydrolytic plasticization and interfacial weakening that enhance lateral plowing and penetration, with late-stage debris compaction moderating further depth growth while width remains high.

**Figure 9:**
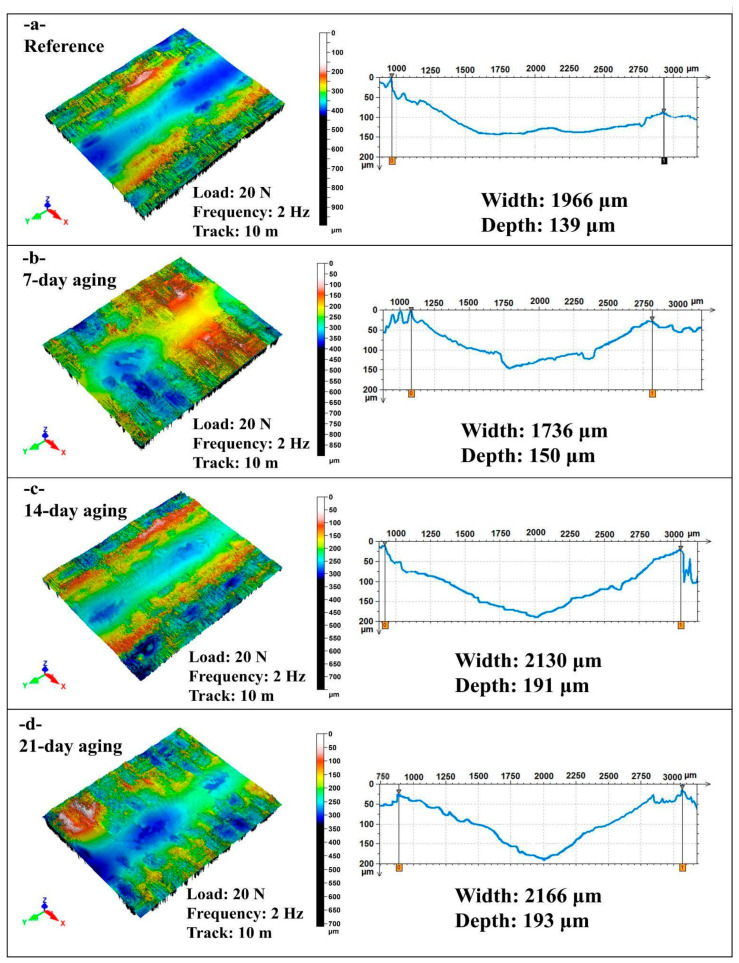
Comparison of width and depth values in reciprocating wear tests at 20 N load, 2 Hz frequency and 10 m sliding distance: (**a**) 0-day aging; (**b**) 7-day aging; (**c**) 14-day aging; (**d**) 21-day aging.

Figure 9 presents non-contact laser profilometry 3D maps and transverse line profiles of reciprocating wear scars for the hybrid epoxy composite tested at 20 N, 2 Hz, and 10 m after 0, 7, 14, and 21 days of water immersion, with wear track width and maximum depth quantified from the profiles.

Figure 9a shows the reference condition with a moderately shallow U-shaped groove, width of 1966 µm and of depth 139 µm, and low symmetric pile-up that indicates limited plastic flow and minimal interfacial failure at test start.

Figure 9b exhibits a narrower but slightly deeper track after 7 days, with a width of 1736 µm, about 12% lower than reference, and a depth of 150 µm, about 8% higher, while the topography reveals a smoother compacted valley consistent with transient moisture-assisted lubrication and debris densification that reduce lateral plowing yet permit modest penetration.

Figure 9c displays a marked enlargement at 14 days, with a width of 2130 µm, about 8% above reference, and a depth of 191 µm, about 37% above reference, with a steeper-walled valley, pronounced pile-up, and micro-scoring that evidence matrix plasticization and fiber–matrix debonding under the higher oscillation rate.

Figure 9d maintains large scars at 21 days, with a width of 2166 µm and depth of 193 µm, while the floor appears smoother and more polished, suggesting damage saturation where continued sliding preserves a broad track and only marginally increases maximum penetration. Water immersion at 2 Hz first produces a short-term narrowing with slight deepening at 7 days due to lubrication and compaction effects, then transitions by 14–21 days to pronounced widening and deepening driven by hydrolytic softening and interfacial degradation, after which depth tends to plateau while the width remains high.

**Figure 10:**
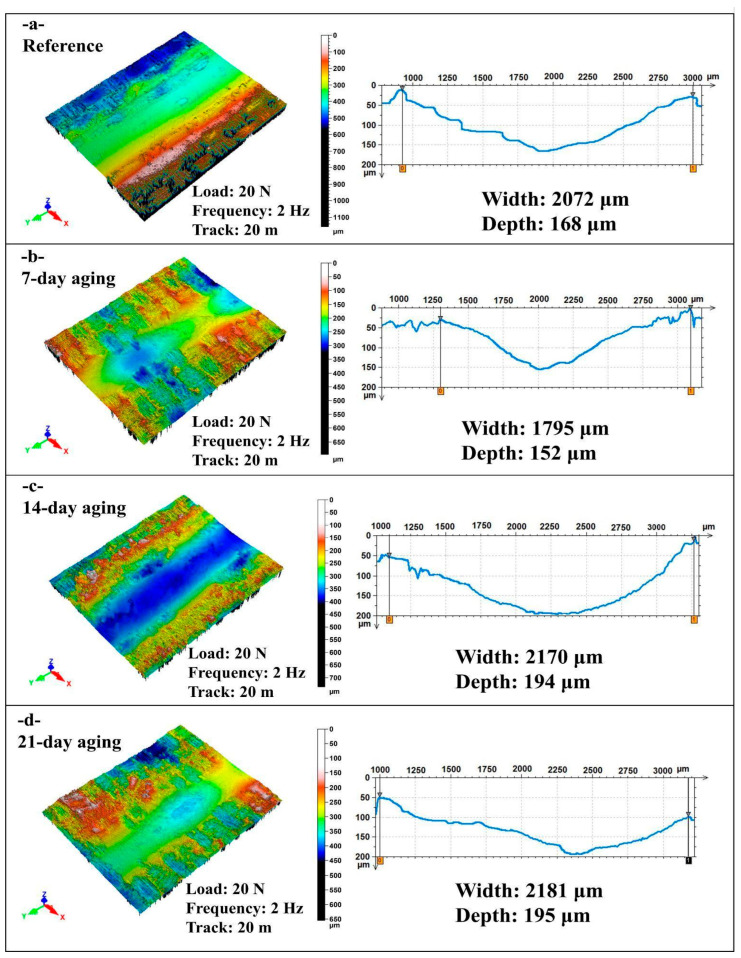
Comparison of width and depth values in reciprocating wear tests at 20 N load, 2 Hz frequency, and 20 m sliding distance: (**a**) 0-day aging; (**b**) 7-day aging; (**c**) 14-day aging; (**d**) 21-day aging.

Figure 10 presents non-contact laser profilometry 3D maps and transverse line profiles for reciprocating scars on the hybrid epoxy composite tested at 20 N, 2 Hz, and 20 m after 0, 7, 14, and 21 days of water immersion, from which wear track width and maximum depth were determined.

In Figure 10a the reference surface shows a broad U-shaped groove with a width of 2072 µm and depth of 168 µm and relatively smooth flanks with limited pile-up, indicating moderate plastic flow and minimal interfacial failure at the start of sliding.

In Figure 10b after 7 days, the track narrows to 1795 µm and depth decreases to 152 µm, roughly 13% and 10% lower than the reference, while the topography displays a smoother and slightly compacted valley, consistent with transient moisture-assisted lubrication and debris densification that reduce lateral plowing and limit penetration.

In Figure 10c by 14 days, the scar expands markedly with a width of 2170 µm and depth of 194 µm, about +5% and +16% versus the reference and roughly +21% and +28% versus 7 days, and the 3D map shows a deeper, steeper-walled valley with pronounced pile-up and micro-scoring that signal matrix softening and fiber–matrix debonding under the higher reciprocation rate and long sliding distance.

Figure 10d at 21-day the track remains wide and deep with a width of 2181 µm and depth of 195 µm, essentially sustaining the 14-day damage level, and the groove floor appears slightly polished which points to damage saturation where debris compaction moderates further deepening while the lateral scar extent stays high.

Water immersion under 2 Hz and 20 m first yields a short-term narrowing and slight shallowing at 7 days that can be attributed to lubrication and compaction effects, followed by pronounced widening and deepening at 14 days that persists at 21 days, indicating a transition to hydrolytic softening and interfacial degradation after which penetration plateaus while lateral plowing remains elevated.

### 3.4. Wear Track SEM Damage Analysis

**Figure 11:**
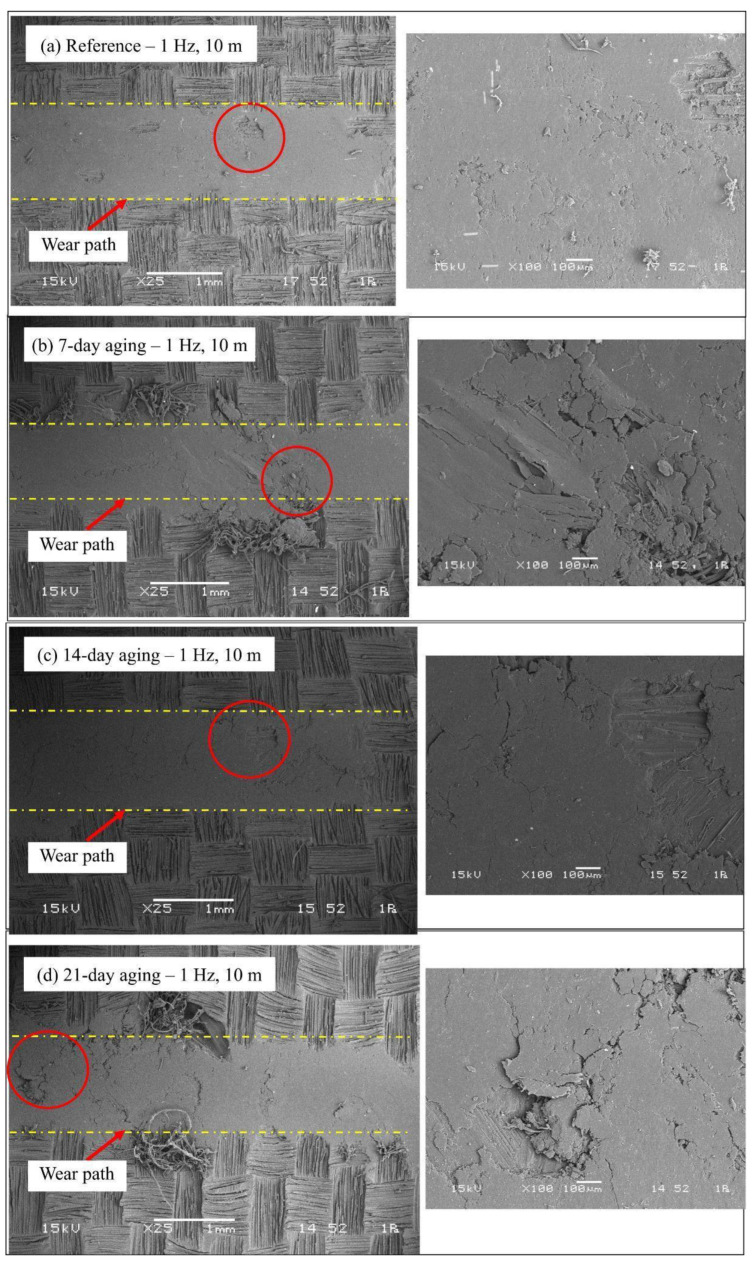
Scanning electron microscopy (SEM) images of reciprocating wear tracks on glass/Kevlar hybrid epoxy composites, tested at a frequency of 1 Hz: (**a**) 0-day aging; (**b**) 7-day aging; (**c**) 14-day aging; (**d**) 21-day aging.

The series of SEM micrographs for the 1 Hz experiments found in Figure 10 presents the gradual degradation of the glass/Kevlar hybrid epoxy composite with an increase in water immersion time. The unaged control sample (Figure 11a) displays an attendant wear track under ambient mild abrasive wear conditions, where matrix plowing is restricted while fiber exposure is minimal, suggesting an undamaged fiber–matrix interface from the load-bearing point of view. After 7 days of exposure to an environment that is hydrothermally aged (Figure 11b), the initiation point for hygrothermal degradation is identified from the enhanced level of matrix micro-cracking coupled with the incipience of fiber-matrix debonding, an intrinsic result emanating from plasticization of the resin allowing enhanced material loss up to the registered increase in wear track width alongside depth. Extensive damage mechanism is initiated after immersing the specimens for 14 days (Figure 11c), where the prevailing hydrolytic tendering coupled with interfacial attenuation has led to widespread fragmentation of the subsequently weakened matrix, substantial debonding, and excessive fiber pull-out, yielding a rough, fractured surface topography consistent with the maximum loss in tribological performance. After 21 days (Figure 11d), the mechanism of damage moves toward a saturation regime portrayed by the formation of an impenetrable packed layer consisting of the wear debris from the partial smoothening of the track floor; the implication is that although the subsurface remains considerably impaired, the continued sliding tenders the deteriorated material by rendering an appreciable restriction to an enormous penetration depth increment even though the continued high-wear track width is maintained.

**Figure 12:**
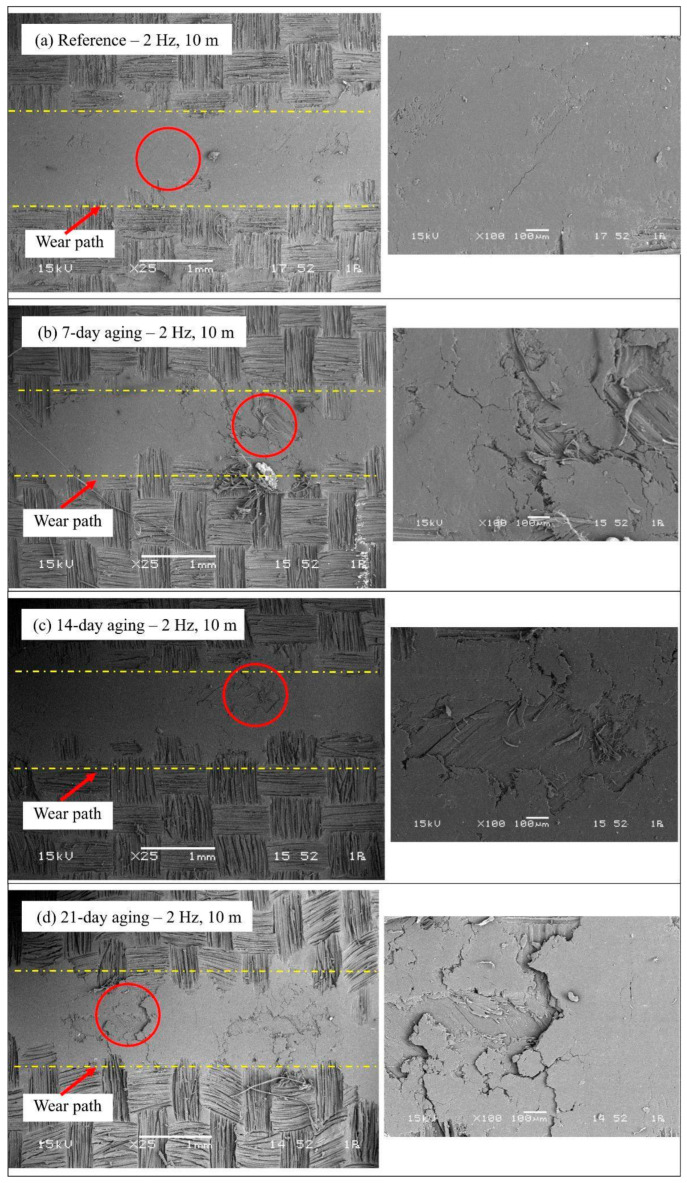
Scanning electron microscopy (SEM) images of reciprocating wear tracks on glass/Kevlar hybrid epoxy composites, tested at a frequency of 2 Hz, (**a**) unaged condition (0 day), (**b**) after 7-day of aging, (**c**) after 14-day of aging, and (**d**) after 21-day of aging.

Moisture governs the tribological response primarily through interfacial processes rather than wear scar geometry alone. Gravimetric uptake up to 8.54% sets the degree of epoxy plasticization and interfacial hydration; at about 14 days a moisture-mediated third body stabilizes at the interface, lowering interfacial shear and producing a pronounced minimum in the steady-state μ. With further immersion, progressive hydrolysis and fiber/matrix debonding increase debris generation and three-body abrasion, which widens and deepens the wear track even as μ partially recovers. Factorial ANOVA ranks immersion time as the dominant factor for both width and depth, with frequency secondary and sliding distance minor, indicating that moisture-controlled interphase integrity, rather than sliding geometry, governs damage evolution. It is important to stress that the observed transient transfer film and the intrinsic wear resistance of the fibers are not mutually exclusive phenomena; rather, their protective effectiveness changes with the state of the matrix and the fiber–matrix interface. Initially (unaged to 7-day) moisture-assisted plasticization mobilizes matrix material and debris which consolidate into a lubricious, water-mediated tribofilm. That film reduces local shear strength and spreads contact over a larger area, thereby temporarily shielding embedded fibers from direct metal contact and lowering COF (Figure 11 and Figure 12). However, the long-term protective effect of that film depends on the integrity of the matrix and the interfacial adhesion: progressive hydrolytic attack (14–21 day) weakens the bond between fibers and resin so that fibers become deboned and protrude or detach. Fibers are exposed; they no longer act as ideal load-bearing reinforcements. They are directly subjected to abrasive three-body action, fibrillation, pull-out, and fracture. In other words, fibers are theoretically more wear resistant than the matrix when fully embedded and bonded, but once exposure and debonding occur their effective resistance can fall sharply because new wear modes (fiber fragmentation and three-body abrasion) dominate. This sequence reconciles the temporary COF reduction and transient narrowing seen at short/intermediate immersion with the dramatic widening, deepening, and catastrophic fiber damage observed at longer immersion.

The SEM micrographs for the 2 Hz reciprocating tests (Figure 12) indicate an evident and faster development of wear damage compared to the 1 Hz case, triggered by the enhanced frictional energy input. The unaged material (Figure 12a) already presents a substantially more damaged surface compared to the 1 Hz one, showing apparent matrix plowing and first fiber ruptures, testifying to an accelerated abrasive-adhesive wear mechanism from the very first. After 7 days of immersion (Figure 12b), there is the onset of an original transient regime characterized by the flattening and densification of the surface layer; this points to the establishment of a stable tribofilm where the plasticization triggered by the moisture, coupled with the elevation of the frequency, encourages the densification of the wear debris, momentarily alleviating the friction and confining the penetration. Unfortunately, this protective action is erased by 14 days of immersion (Figure 12c), where the surface presents catastrophic degradation. The micrograph exhibits wide distribution of the matrix ripping, heavy fiber fragmenting, and extensive fiber pull-out, caused by the combined action of progressed interfacial hydrolysis and the strong cyclic stressing by the frequency up to 2 Hz. The damage by 21 days (Figure 12d) is up to the saturation level, showing an inconsistent morphology consisting of deep grooves that are plowed away, exposed bundles toward the surface, and an inconsistent distribution consisting of packed-down regions mixed up with recently broken-down ones, highlighting an autonomous cycle material elimination overruling the stability of the compaction action by the most severe combination of environmental and mechanical inputs.

The SEM images therefore must be read in the context of concurrent chemical and mechanical transformations triggered by water immersion. Chemically, absorbed water initiates two linked processes: (i) reversible plasticization of the epoxy network through hydrogen bonding to polar groups (reducing local glass transition and stiffness) and (ii) slower hydrolytic scission of susceptible bonds at the fiber–matrix interface and within the near-surface resin leading to irrevocable interfacial weakening. Mechanically, plasticization lowers the matrix’s cohesive strength and promotes microcrack nucleation under cyclic shear, while hydrolysis and interfacial debonding reduce interlaminar shear transfer so that fibers progressively relocate from a load-bearing to a loose/exposed state. On the SEM this converts early-stage matrix plowing and compacted transfer-film appearance (7 days) into later-stage signatures of interfacial failure with widened grooves, ragged exposed fibers, fibrillation, fiber fragmenting and pull-out (14–21 days). In addition, the compacted debris layer seen at the longest exposures reflects a mechanical saturation where three-body abrasion and debris compaction compete: the debris both protects (by filling the groove) and abrades (by acting as hard third bodies) depending on its composition and consolidation state. The wear mechanisms deciphered by the SEM micrographs in Figure 11 and Figure 12 are very much reinforced by evidence from the current published research materials on hygrothermal degradation for polymer-type composites. The noticed migration from frail abrasion when still unaged to harsh fragmentation from the resin, coupled with the interfacial degradation by 14 days’ immersion, is the direct outcome from the plasticizing resin coupled with the interfacial degradation, an well-reported phenomenon by the research material conducted on the epoxy type material under the influence of water aging [18]. In addition, the novel transient formation of the smoothed and compacted tribofilm present at 7 days for the 2 Hz case (Figure 12b) is consistent with reported hydrothermally triggered boundary lubrication capable of transiently diminishing wear and friction, as reported for multi-filler epoxy composites under hygrothermal loading [23]. Lastly, the catastrophic debonding of the fibers from the matrix and the saturation behavior with increased immersion time are consistent with processes where progressive hydrolytic weakening of the fiber–matrix interface catastrophically leads to dominance by three-body abrasion and loss of structural integrity, consistent with reported tribological studies on aged fiber-reinforced composites [24].

**Figure 13:**
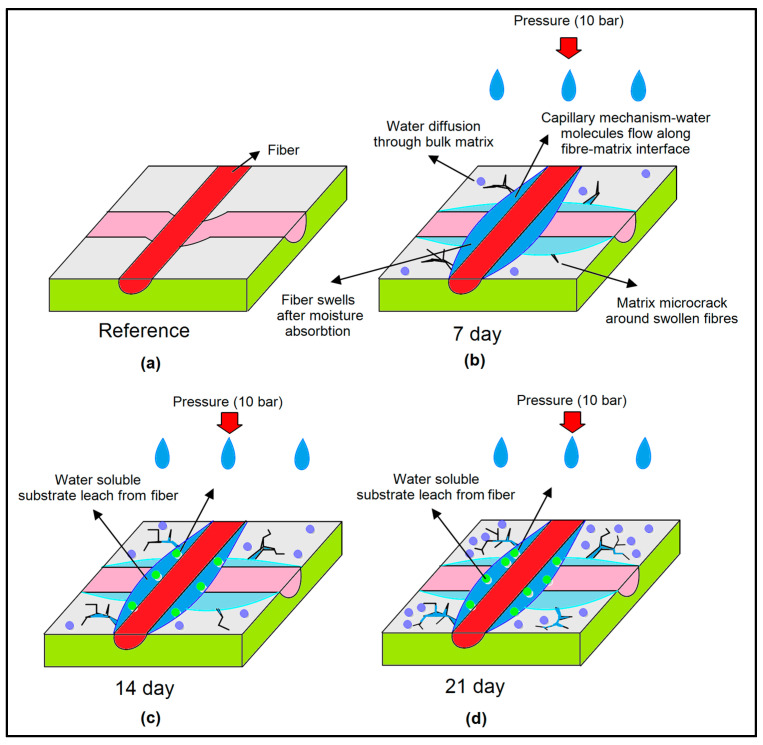
Moisture-driven degradation mechanism under 10 bar immersion and its tribological effect: (**a**) 0-day intact interface, stiff matrix, narrow wear track; (**b**) 7-day initial water ingress, local plasticization, early transfer layer; (**c**) 14-day strong debonding and fiber pull-out, wide/deep wear scar, low-friction tribofilm; (**d**) 21-day near-saturation, three-body abrasion by debris, unstable tribolayer, partial friction recovery.

Figure 13 illustrates the moisture-induced gradual degradation mechanism of the hybrid composite when immersed in water at 10 bar pressures. As can be seen, water uptake at 7 days, 14 days, and 21 days altered the fiber–matrix interface, matrix integrity, and wear scar morphology. The diagram relates these microstructural changes to the measured tribological response, demonstrating that interface swelling, plasticization, debonding, tribofilm formation, and finally, three-body wear control both the coefficient of friction and wear severity.

In the unaged state, in Figure 13a, the glass/Kevlar hybrid epoxy laminate shows full interfacial integrity and the epoxy matrix remains in high-stiffness condition. The woven fibers are well anchored and there is no evidence of matrix microcracking or fiber to matrix debonding in the wear track. The reciprocating wear scar at this stage is shallow and moderately narrow, which indicates limited plastic flow and minimal interfacial failure. During sliding the load is transferred efficiently across the intact fiber to matrix boundary so local stiffness and interlaminar shear strength remain high.

In Figure 13b during 7-day immersion under 10 bar pressurized water, the sample is in the transient uptake regime in which water diffuses through the epoxy matrix and preferentially along the fiber to matrix interface. Capillary pathways at locally hydrophilic Kevlar-rich regions promote local matrix plasticization and swelling around the fibers. This initiates microcracks and the first stages of interfacial debonding. Mechanically the interfacial shear strength and effective near-surface stiffness begin to drop because the matrix can no longer clamp the fibers as rigidly as in the dry state. The slightly softened moisture-plasticized matrix and fine debris begin to smear and compact in the reciprocating groove, and this produces an early hydrated transfer layer. The compacted debris layer distributes contact stress more uniformly which can transiently suppress stick–slip and partially lower friction relative to the dry reference.

In Figure 13c during 14-day immersion representing intermediate quasi-equilibrium swelling, the laminate has absorbed substantially more water. The epoxy is now significantly plasticized and fiber to matrix interfaces are partially hydrolyzed and weakened. The reciprocating wear scar becomes markedly wider and deeper which reflects lateral plowing and easy penetration into the softened and poorly supported interphase. At the same time this heavily plasticized debris rich surface compacts into a coherent hydrated tribofilm that acts as a low shear third body. Fourteen day immersion therefore marks the stage where mechanical integrity is strongly degraded with loss of stiffness and interfacial cohesion geometric wear is severe with track widening and deepening, but tribological behavior is paradoxically most favorable in terms of friction reduction because a stable lubricious tribolayer is present.

In Figure 13d during 21-day immersion representing near saturation and degradation saturation, the laminate approaches quasi-saturation of water uptake. The matrix is extensively plasticized and fiber to matrix adhesion is strongly compromised, and widespread interfacial debonding fiber exposure and resin fragmentation are observed. Only marginal additional deepening occurs which indicates a damage saturation state in which further penetration is limited by debris compaction and polishing of the groove. At 21 days the laminate is therefore both mechanically compromised with a largely collapsed interphase and tribologically less efficient than the 14 -day state showing higher and more erratic friction despite the already widened and stabilized wear scar geometry.

### 3.5. Factorial Contribution Analysis

Factorial analysis is a comprehensive statistical method employed to quantify the relative contribution and interaction of multiple experimental factors on some measured response. In engineering and materials science, it is a widely used tool for identifying factors that significantly affect performance by determining which process parameters have significant effects on performance outputs, allowing systematic optimization instead of running on a trial-and-error basis. By breaking the total variance in the experimental outcomes into the contributions of individual factors as well as their interactions, the factorial analysis provides the quantitative foundation on the complicated cause–effect relationships under multi-parameter systems.

In the present work, factorial contribution analysis was utilized in assessing the contributions of significant test parameters—immersion days, sliding frequency, and track length—on hybrid glass/Kevlar epoxy composite wear responses. Of particular interest was the determination of the contributions of these factors towards variability in scratch depth and scratch width, which are significant indicators of tribological degradation. Beyond identifying main effects, the method also estimates factor interactions (synergistic or antagonistic). In order to ensure statistical robustness and incorporation of reproducible patterns, the analysis was conducted on data obtained from three separate independent experimental sets, thus providing a robust basis on which the spontaneous tribo-decorators’ wear could be interpreted under varied hygrothermal as well as mechanical conditions.

Table 2 presents the general factorial regression results obtained to establish the relative experimental parameter contribution towards the scatter in scratch width values. It consists of the following factors: immersion days, reciprocating frequency, and track length, along with the two- and three-way interactions among the factors. The regression model accounted for 99.85% of the total variance, establishing a very reliable correspondence between the experimental data and the statistical model. Out of the individual factors, the immersion duration (Days) has the highest influential character, contributing the highest towards the total variation, which is 73.69%, followed by frequency (12.99%) and track length (0.96%). High values of significant F-values and *p*-values (*p* < 0.05) for all the primary factors further establish their statistical relevance. Interaction terms, especially Days × Frequency, also indicate a significant contribution (11.82%), providing evidence towards the existence of a synergy between the time of exposure and the sliding frequency on the scratch width. The low error contribution (0.15%) indicates uniform data and supports the model’s predictive capability.

**Figure 14:**
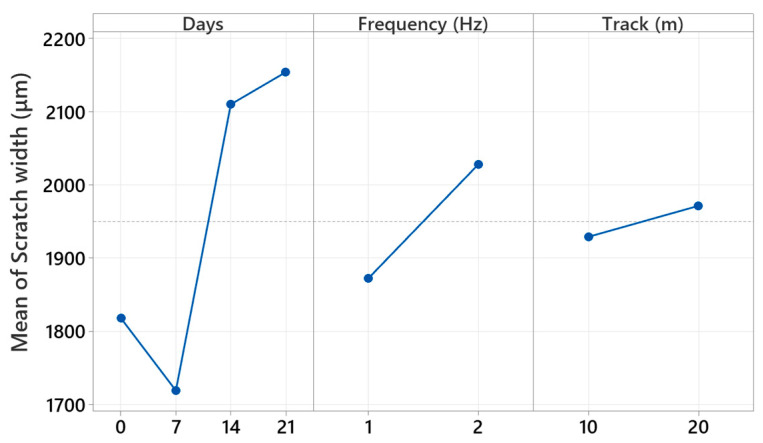
Main effect plots of immersion days, reciprocating frequency, and track length on the scratch width of hybrid glass/Kevlar epoxy composites.

Figure 14 presents the main effect plots from the factorial analysis, which represent the autonomous effect of each test parameter on the measured scratch width. The plots indicate that immersion time has the highest impact, and increasing the scratch width is significant, as the aging time goes from 0 to 14 days and levels off towards the end at 21 days. This trend is representative of the hydrolytic softening and interface degradation reactions that occur in the composites, causing the wear tracks to be wider. The frequency factor also presents a positive trend, such that the increasing the reciprocating frequency (2 Hz) produces the wider scratch due to the increased frictional energy input and raised dynamic contact stresses. Conversely, the track length presents a generally minor but consistent effect, such that increased sliding extends the surface deformation through a cumulative material removal process. Overall, the main effect plots validate that immersion time is the overwhelming parameter that determines surface damage, and the frequency and track length serve as the second-order contributors determining the extent and morphology of the wear response.

Table 3 shows the general factorial regression results carried out for assessing the relative importance of immersion time, reciprocating frequency, and track length on the hybrid glass/Kevlar epoxy scratch depth. The regression model yields high determination coefficients, explaining 99.70% of the total variability, signifying high association between the factors and the experimental results. Among the linear terms, immersion days display the highest effect, contributing 87.96% towards the total contribution, followed by frequency (4.96%) and track length (1.67%). These outcomes imply that increased water immersion substantially deepens the scratch penetration owing to the softening of the matrix and weakening of the interface, whereas increased frequencies augment the mechanical degradation owing to enhanced local stresses and heat production. Two-way and three-way interaction terms, specifically Days × Frequency, are found statistically significant (*p* < 0.05) as well, signifying that the combined environmental as well as the effects of the mechanics work towards the enhancement of wear severity in a synergistic fashion. Very low error fraction (0.30%) further validates the robustness and the reproducibility of the data. In general, the factorial regression outcomes validate that the immersion time is the leading factor that affects the scratch depth, followed by significant, though lower, effects by the reciprocating frequency as well as the sliding distance.

**Figure 15:**
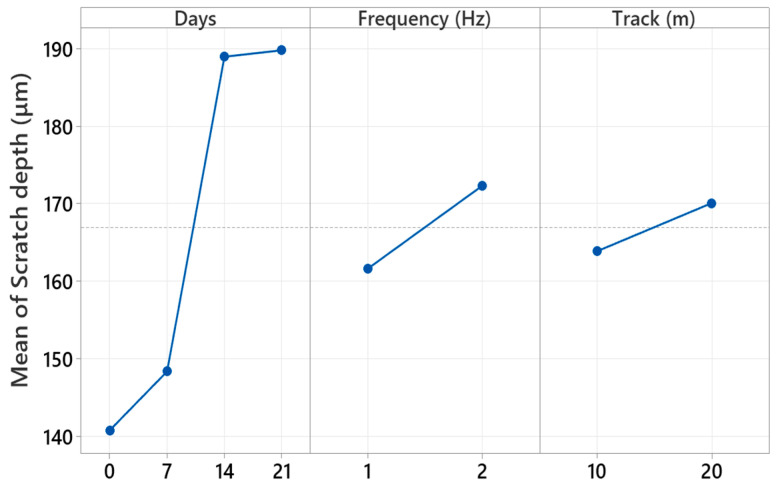
Main effect plots of immersion days, reciprocating frequency, and track length on the scratch depth of hybrid glass/Kevlar epoxy composites.

Figure 15 presents the principal effects of immersion time, reciprocating frequency, and track length on the scratch depth, as obtained from the factorial regression analysis. Trends in the plots indicate that the immersion days have the greatest effect on scratch depth. A significant increase is observed up to 14 days of submerged time in water, equivalent to hydrolytic softening of the epoxy matrix and sequential fiber/matrix debonding, allowing deeper material infiltration on sliding. After the saturation point at more than 14 days, the depth values tend towards equilibrium, suggesting a saturation phase or phase when surface degradation and debris hardening would level further penetration. Also, the reciprocating frequency is found using a reasonable effect test to by and large have deeper scratches at 2 Hz compared to testing at 1 Hz, due to high frictional heat generation and cyclic inversion of the stressing concentrations. However, the track length is found with a relatively small effect, showing a moderate increase in depth with increasing sliding distance due to the build-up of the wear and surface fatigue. Overall, the results validate that the time of immersion is the key determinant that governs the evolution of the subsurface damage, whereas the reciprocating frequency and track length are the moderators that control the extent of the deformation induced by the wear.

**Figure 16:**
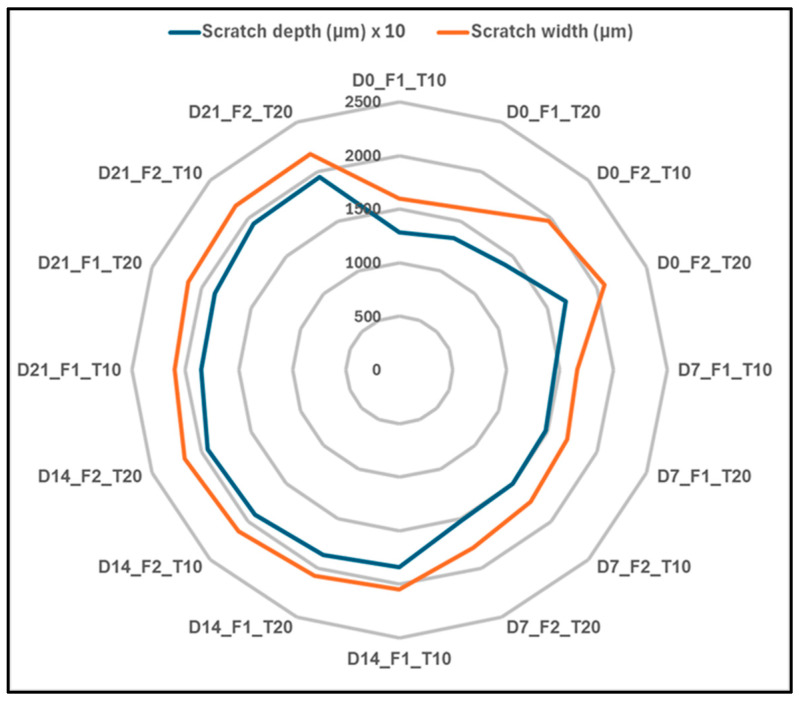
Radar graph of the factorial contribution ratios of immersion days (D), reciprocating frequency (F), and track length (T) on scratch depth and scratch width of hybrid glass/Kevlar epoxy composites.

Figure 16 is the radar plot showing the relative factorial impact on the following parameters: immersion days (D), reciprocating frequency (F), and track length (T), by the scratch depth and scratch width variability. The radar plot summarizes the relative influence of each factor on the overall visualization of the influence from each factor on the wear-related surface property of the hybrid composite system. The distribution on the radar is very clear such that the immersion time (D) is the leading one on both scratch width and depth, creating the longest axis on the plot. This leading is a clear indication of the association between the hydrothermal conditioning and the breakdown on the interfacial bonding in the epoxy matrix, which controls the level on the surface and subsurface deformation.

Reciprocating frequency (F) also presents a high, second-order effect, specifically apparent in the scratch width measurements, showing that increased sliding speed amplifies frictional energy dissipation and stress, hence increasing surface deterioration. Track length (T) is, however, a minor contributory factor towards the combined variation, meaning that increased distance between slides has little impact on altering the underlying wear mechanism, rather influencing the cumulative wear. The radar plot thereby summarizes the percentage hierarchy of factor influences, verifying the immersion days as the chief cause of wear severity, with frequency and track length acting as supplementary parameters that vary the intensity of the damage under varied test conditions.

## 4. Conclusions

Hybrid basalt/carbon laminates subjected to reciprocating sliding in moist and aqueous environments exhibit a moisture–interphase–third-body pathway that governs performance more than wear-track geometry alone. Immersion time emerges as the dominant variable shaping both friction and damage, with loading frequency secondary and sliding distance of lesser influence; together these factors define a moisture-controlled operating window. By deriving specific wear and frictional-energy metrics from existing data, this study shows that water controls tribology via third-body and interphase changes that can reduce μ while simultaneously enlarging damage, resolving the apparent contradiction between low friction at 14 days and increased wear cross-section.

At intermediate exposure, moisture reorganizes the interfacial third body and lowers interfacial shear, leading to a stable reduction in steady-state μ. With extended immersion, hydrolytic softening and progressive fiber/matrix debonding intensify debris-assisted three-body abrasion, widening and deepening the wear cross-section even as friction partially recovers. Profilometry and microscopy thus corroborate a friction–damage decoupling; the system can slide more easily while accumulating greater geometric loss, because interphase integrity, not merely contact kinematics, sets the trajectory of damage evolution.

These findings offer actionable value for components operating in humid, splash, or submerged service. Maintenance and inspection schedules should be keyed to immersion exposure rather than sliding distance alone, particularly avoiding prolonged pre-soak that pushes the system into the high-damage regime. Practical mitigation pathways include barrier coatings or sealants to limit uptake, interphase toughening and sizing/coupling strategies to resist hydrolysis, and fiber-surface treatments that stabilize transfer-film coherence so that low friction can be retained without sacrificing wear resistance.

Building on this mechanistic picture, future work should systematically map temperature–humidity–pressure–salinity matrices to generalize the identified design window, and develop interphase-engineering routes (nanoparticulate interlayers, optimized sizing, or advanced coupling agents) that sustain third-body stability under moisture. In situ or operando tribometry, combined with spectroscopic tracking of transfer-film chemistry, will clarify transition points between lubricious and abrasive regimes. Finally, predictive life models that couple diffusion, plasticization, and interfacial debonding with reciprocating contact mechanics are needed to translate coupon-scale insights to component-level durability and to guide materials selection and duty-cycle optimization in wet service.

## Figures and Tables

**Table 1:** Reciprocating wear test parameters for composites after 0-day, 7-day, 14-day and 21-day water immersion.

Reciprocating Parameters			
No	Force	Frequency (Hz)	Track (m)
1	20 N	1	10
2	20 N	1	20
3	20 N	2	10
4	20 N	2	20

**Table 2:** General Factorial Regression: Scratch width (μm) versus Blocks; Days; Frequency (Hz); Track (m).

Source	DF	Seq SS	Contribution	Adj SS	Adj MS	F-Value	*p*-Value
Model	17	2,245,494	99.85%	2,245,494	132,088	1155.86	0
Blocks	2	107	0.00%	107	54	0.47	0.631
Linear	5	1,970,858	87.64%	1,970,858	394,172	3449.28	0
Days	3	1,657,320	73.69%	1,657,320	552,440	4834.25	0
Frequency (Hz)	1	292,032	12.99%	292,032	292,032	2555.49	0
Track (m)	1	21,505	0.96%	21,505	21,505	188.19	0
2-Way Interactions	7	272,800	12.13%	272,800	38,971	341.03	0
Days × Frequency (Hz)	3	265,901	11.82%	265,901	88,634	775.61	0
Days × Track (m)	3	5719	0.25%	5719	1906	16.68	0
Frequency (Hz) × Track (m)	1	1180	0.05%	1180	1180	10.33	0.003
3-Way Interactions	3	1729	0.08%	1729	576	5.04	0.006
Days × Frequency (Hz) × Track (m)	3	1729	0.08%	1729	576	5.04	0.006
Error	30	3428	0.15%	3428	114		
Total	47	2,248,922	100.00%				

**Table 3:** General Factorial Regression: Scratch depth (μm) versus Blocks; Days; Frequency (Hz); Track (m).

Source	DF	Seq SS	Contribution	Adj SS	Adj MS	F-Value	*p*-Value
Model	15	27,684.1	99.70%	27,684.1	1845.61	697.55	0
Linear	5	26,263.3	94.58%	26,263.3	5252.65	1985.26	0
Days	3	24,424.7	87.96%	24,424.7	8141.58	3077.13	0
Frequency (Hz)	1	1376	4.96%	1376	1376.02	520.07	0
Track (m)	1	462.5	1.67%	462.5	462.52	174.81	0
2-Way Interactions	7	1109.8	4.00%	1109.8	158.54	59.92	0
Days × Frequency (Hz)	3	647.6	2.33%	647.6	215.85	81.58	0
Days × Track (m)	3	420.1	1.51%	420,1	140.02	52.92	0
Frequency (Hz) × Track (m)	1	42.2	0.15%	42.2	42.19	15.94	0
3-Way Interactions	3	311.1	1.12%	311.1	103.69	39.19	0
Days × Frequency (Hz) × Track (m)	3	311.1	1.12%	311.1	103.69	39.19	0
Error	32	84.7	0.30%	84.7	2.65		
Total	47	27,768.8	100.00%				

## Data Availability

The datasets presented in this article are not readily available because the data are part of an ongoing study. Requests to access the datasets should be directed to the corresponding author.

## Abbreviations

The following abbreviations are used in this manuscript:

ANOVA	Analysis of Variance
SEM	Scanning electron microscope
